# Factors influencing the current practice of self-medication consultations in Eastern Indonesian community pharmacies: a qualitative study

**DOI:** 10.1186/s12913-016-1425-3

**Published:** 2016-05-13

**Authors:** Cecilia Brata, Colleen Fisher, Brahmaputra Marjadi, Carl R. Schneider, Rhonda M. Clifford

**Affiliations:** Centre of Medicine Information and Pharmaceutical Care, The University of Surabaya, Surabaya, Indonesia; Pharmacy, School of Medicine and Pharmacology, The University of Western Australia, Perth, Australia; School of Population Health, The University of Western Australia, Perth, Australia; School of Medicine, The University of Western Sydney, Campbelltown, Australia; Faculty of Pharmacy, The University of Sydney, Sydney, Australia

**Keywords:** Self-medication consultations, Community pharmacies, Indonesia

## Abstract

**Background:**

Research has shown that the current practice of pharmacy staff when providing self-medication consultations in Indonesia is suboptimal. To improve the performance of pharmacy staff when providing self-medication consultations in community pharmacies, the factors that influence current practice need to be understood. The aim of this study is to identify the factors that influence current practice of pharmacy staff when handling self-medication consultations in Eastern Indonesian community pharmacies.

**Methods:**

Fifteen in-depth interviews were conducted with pharmacists, pharmacy technicians, pharmacy owners, and counter attendants. Thematic analysis was used to generate findings.

**Results:**

The current practice of pharmacy staff when handling self-medication consultations is directly influenced by the professionalism of pharmacy staff and patient responses to the consultations. These factors are in turn affected by the organisational context of the pharmacy and the external pharmacy environment. The organisational context of the pharmacy includes staffing, staff affordability, and the availability of time and facilities in which to provide consultations. The external pharmacy environment includes the number of trained pharmacy staff in the research setting, the relevance of pharmacy education to the needs of pharmacy practice, the support offered by the Indonesian Pharmacists Association, a competitive business environment, and the policy environment.

**Conclusion:**

Complex and inter-related factors influence the current practice of pharmacy staff when providing self-medication consultations in community pharmacies in this research setting. Multiple strategies will be required to improve consultation practices.

**Electronic supplementary material:**

The online version of this article (doi:10.1186/s12913-016-1425-3) contains supplementary material, which is available to authorized users.

## Background

Self-medication is defined as “the selection and use of medicines by individuals to treat self-recognized illnesses or symptoms” [1]. In Indonesia, self-medication is highly prevalent: 91 % of Indonesians were reported as practicing self-medication in 2013 [2]. To self-medicate, patients often obtain medicines from community pharmacies, with the 2013 National Health Basic Research showing that the majority of households in Indonesia purchased their medicines through community pharmacies [3, 4]. Community pharmacies, therefore, have the potential to make a significant contribution to the health care of the public by providing appropriate advice for patients with self-medication requests [4].

Despite the potential contribution that community pharmacies could make to the health of Indonesians, the performance of pharmacy staff when providing self-medication counselling in Indonesia is reported as being poor [5–8]. Information-gathering performed by pharmacy staff is not comprehensive, the advice provided to patients is often inappropriate, and most pharmacy staff fail to provide information regarding the medication sold [5–8]. As research has shown that providing appropriate advice when handling self-medication requests in community pharmacies can improve patient outcomes [9–11], it is important to improve the quality of counselling for self-medication in Indonesian community pharmacies.

Any improvements in community pharmacy services for self-medication must take into account the health needs of the patients, the health-care system, and the socio-cultural context [12]. To be able to design a sustainable intervention model that will best fit with the local context, it is important to understand the factors that influence the current practice of pharmacy staff when handling self-medication requests. The aim of this study, therefore, is to identify the factors that influence the current practice of pharmacy staff when handling self-medication consultations in Eastern Indonesian community pharmacies.

## Methods

### Research setting

This qualitative study was conducted in community pharmacies in a provincial capital located in the eastern part of Indonesia. The research site had a population of approximately 390,000 in 2013 [13, 14]. There are more than 250 indigenous and 100 non-indigenous groups residing in this area [15], attesting to the multicultural context in which the research was undertaken. In Indonesia, development has not been uniform between areas; the research setting is considered to be one of the least developed areas of Indonesia [16]. Health indicators for the area are poor. In 2012, the infant and under-5 mortality rates were 54 and 115 per 1000 births respectively. In comparison, the rates for Jakarta, the capital city of Indonesia, were 22 and 31 respectively [17]. There is a shortage of trained pharmacy staff (i.e., pharmacists and pharmacy technicians) in the workforce in this setting [17]. Currently, three pharmacy technician schools are located in the setting. Two of these schools were recently opened; to date, only one school has produced graduates. There is no university in this area that currently has a pharmacist program.

### Sample and recruitment

Participants were purposively sampled to gain a diverse range of perspectives. Prior to this qualitative study, the researcher (CB) conducted a quantitative survey of the demographic characteristics of pharmacies and pharmacy staff, personally visiting all pharmacies in the research setting. Rapport with participants, therefore, had previously been established. Based on the data collected from the quantitative survey, 15 pharmacy staff were initially selected. The selection of participants was based on a range of characteristics: their role in the pharmacy, their age, and the size of the pharmacy. The first author (CB) contacted all participants either by phone or in person and provided information about the study. If participants agreed to participate, an interview place and time was arranged. If a selected participant refused, another person with similar characteristics was contacted. It was planned that recruitment would continue until data saturation was achieved.

### Data collection

Before the interviews started, the interviewer (CB) explained the purpose and scope of the study and sought written, informed consent to participate. Face-to-face, in-depth interviews were aided by an interview guide (see Additional file 1), that was prepared by the first author (CB), reviewed by two co-authors (CF and RM), and refined as interviews progressed. Of the total 15 interviews, 14 were audio-recorded. Extensive notes were taken for the remaining interview because the participant refused to be audio-recorded. Interviews were recursive in nature: the interviewer often restated or summarised information and sought feedback from the participant that appropriate interpretations of the data were being made [18]. All interviews were conducted in the Indonesian language by the first author (CB), whose first language is Indonesian. The interviews lasted between 30 and 60 min.

### Data analysis

Data collection and data analysis were undertaken simultaneously. All interviews were transcribed in the Indonesian language and thematic analysis as described by Braun and Clarke was used to inform data analysis [19]. The data analysis was performed by the first author (CB) and the emerging themes were discussed with co-authors. The data analysis firstly involved a process of familiarisation with the data by listening to the audio-recordings and reading the transcripts several times and noting ideas. Following this, significant statements relating to factors that influenced the current practice of self-medication consultation from the data were identified and coded. These codes were then clustered and organised at a broader conceptual level (i.e., into themes). The process of theme generation was reviewed and refined by going back and forth between the themes and the codes, as well as between the themes and the transcripts until the final themes were identified, defined, and named. The final themes were discussed with a group of pharmacists who participated in the study in a member-checking process. Finally, examples of responses were selected to illustrate each theme. The data analysis was conducted in Indonesian and the illustrative verbatim quotes and themes labels were translated into English by the first and the third author (CB, BM). NVivo7 software (QSR International) was used to facilitate data analysis, and for data storage, management, and interrogation. The study was approved by the Human Research Ethics Committee of the University of Western Australia and the local Indonesian Pharmacists Association.

## Results

Fifteen pharmacy staff were interviewed (Table 1). Data saturation occurred after the twelfth interview, from which no new information on factors influencing current self-medication consultation practice was gained during data analysis. However, as a further three participants had already been contacted and agreed to participate in the study, all 15 participants were included.

**Table 1:** Demographic profile of participants

Positions in the pharmacy	n	Age range (years)	Experience (years)	Size of pharmacy
Pharmacist	9	26–55	2–20	• small (3 participants)
				• medium (4 participants)
				• large (2 participants)
Pharmacy technician	2	22–37	3–5	• small (1 participant)
				• medium (1 participant)
Pharmacy owner	2	50–56	6–30	• small (1 participant)
				• large (1 participant)
Counter attendant	2	40–41	2–20	• small (1 participant)
				• large (1 participant)

• Small pharmacies were defined as pharmacies with a reported total number of patients of less than 50 per day. Such pharmacies were typically independent pharmacies, unattached to a doctors’ clinic

• Medium pharmacies were defined as pharmacies with a reported total number of patients between 51 and 99 per day. Such pharmacies usually were attached to a doctors’ clinic that serviced a relatively small number of patients

• Large pharmacies were defined as pharmacies with a reported total number of 100 or more patients per day. Such pharmacies were usually attached to a busy doctors’ clinic

**Fig. 1:**
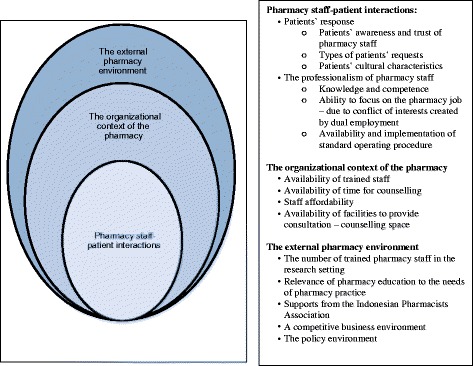
Factors related to the provision of self-medication consultation in community pharmacies in this setting

Whether and how pharmacy staff provided self-medication consultations in this setting was influenced by factors that related to three main themes, namely: (1) pharmacy staff-patient interactions; (2) the organisational context of the pharmacy; and (3) the external pharmacy environment. The identified themes were complex and intertwined and, as such, an ecological model was adapted to explain the interconnections [20, 21]. This model features multiple levels of influence, which are interactive and reinforcing (Fig. 1) [22, 23].

### Pharmacy staff-patient interactions

Interactions between pharmacy staff and patients during the consultation process were related to patients’ responses and the professionalism of pharmacy staff.

#### Patients’ responses

Participants perceived that patients did not always provide an appropriate response when pharmacy staff asked for their medical information or when their medications were explained. As a pharmacy technician mentioned: *“Actually we want to ask the patients, but … it seems like the patients don’t want to be interrogated.”* A pharmacist also stated: *“I am actually very happy to provide information to patients if their responses are positive. However, in the situation where patients do not care, it is useless to provide the information.”* Patients’ responses might be influenced by their awareness and trust of the role and competence of pharmacists, their types of requests, and their cultural characteristics.

Pharmacists perceived that patients might not be aware of their role and competence and did not place as much trust in them as they did in doctors. A pharmacist commented: *“I think our community does not know that we have had formal education in pharmacy. The community thinks that we are just high school graduates who then received a bit of training to work at a pharmacy.”* Another pharmacist mentioned: *“For some cases, I always ask our doctor [who practises here] to explain to patients. Patients trust doctors more. If we are the ones who explain it to them, they are not always willing to listen.”* Therefore, the data suggests that patients might not perceive pharmacists as health-care professionals.

The types of requests made by patients were also considered to influence interactions between pharmacy staff and patients. Participants perceived that patients responded more positively to questioning when they came to the pharmacy with symptom-based requests; and hence, reported better engagement with patients who came with symptom-based requests than with those with product-based requests. Patients who came to the pharmacy with product-based requests usually wanted simply to purchase their medicines and did not like to be questioned. Lower levels of consultation for patients with product-based requests may also be due to a perception by pharmacy staff that such patients are already familiar with the products they request, as stated below:

*“If patients arrive and immediately ask for a drug, it means they already know what they want, so I don’t ask any questions. But if patients arrive and ask questions about their sickness … they don’t know what drug they want … in that case I will ask [questions].”* (counter attendant)

Patients’ cultural characteristics were also perceived as affecting their responses in self-medication consultations. Pharmacy staff noted that these characteristics were different in this setting to other places in Indonesia:

*“Patients here are different from patients in Java [the most developed area in Indonesia]. In Java, we are still able to inform patients and they listen [to us]. Here if a patient isn’t willing to listen, they won’t listen. Patients here are more strong-headed.”* (pharmacist)

In addition, while not a universal view, some non-indigenous participants perceived their indigenous patients to be more temperamental than non-indigenous patients, making the consultation process more difficult. One pharmacist reported: “*It is harder [to provide explanations] to the indigenous people. They are more bad-tempered [than non-indigenous].”*

#### The professionalism of pharmacy staff

The interviews revealed some questionable professional practices. For example, information-gathering as part of the consultation process was not comprehensive. Information about patients’ medical histories and currently used medications were rarely gathered by pharmacy staff when handling self-medication requests. There were also inconsistencies in the provision of the services, as admitted by a pharmacy technician: *“It [whether I provide medicine information to patients] depends on my mood. If I am in the [good] mood, I will provide the explanation. If I am not [in a good mood], I do not provide it.”* Furthermore, pharmacy staff often took a passive stance, in which the interaction was largely driven by the patients. A pharmacist said *“In our current practice, the tendency is that the pharmacy staff wouldn’t ask or provide information if the patients do not ask [questions] first.”* Professionalism in this paper is defined as an individual’s competencies, attitudes, and behaviours appropriate to a particular profession [24, 25]. Problems with professionalism in the research setting appear to be the consequences of a lack of knowledge and competence, an inability of the majority of trained staff to focus on their pharmacy jobs due to dual employment, and a lack of awareness and implementation of national standard operating procedures for self-medication consultations. These three areas are expanded on below.

Currently, self-medication consultations are handled by both trained and non-trained staff. While it was apparent that non-trained staff do not have the competence to undertake self-medication consultations, many pharmacists confessed that they too lack the knowledge and skills with which to provide quality self-medication consultations, as illustrated below:

*“Actually, we need to ask those questions [questions about medical history and current medications being used by patients]. However, the problem is knowledge. For example, I don’t know if this medication interacts with this [other] medication. To be honest, I don’t know. That’s why I say the problem is knowledge.”* (pharmacist)

*“Our knowledge about disease is lacking. We don’t read much and we don’t follow the developments [in new knowledge].”* (pharmacist)

Additionally, problems with professionalism appear to result from the situation in this research setting where the majority of pharmacists work two jobs concurrently: many work in both a government office and in a pharmacy. This double role creates a conflict of interest for pharmacists that affects their professionalism, particularly in terms of their ability to focus on their pharmacy work, as illustrated by the following quotes:

*“Pharmacists have other jobs. They work as government employees [in the morning]… and work at pharmacies in the evening. However, they are already tired by the evening, so they rarely go to their pharmacy jobs.”* (pharmacy owner)

*“It is hard to improve my capacity in my current situation, which is working in a government office and a pharmacy. My concentration goes more to my job in the government office because there are a lot of targets that have to be fulfilled there.”* (pharmacist)

Finally, although the Indonesian Department of Health has published guidelines that contain a standard operating procedure for responding to self-medication requests [26], it appeared that most participants were either unaware of it or had not read it and, as such, it was not implemented.

### Organisational context of the pharmacy

Factors related to the internal pharmacy organisational context were identified as trained staff availability, time for counselling, staff affordability, and facilities to provide counselling.

#### Trained staff availability

Participants reported that it was difficult to find full-time pharmacists and pharmacy technicians to fill the positions available. There is a shortage of trained pharmacy staff in this province of Indonesia, and the majority of those that can be found apparently prefer to work as government employees, as such work is perceived to be more prestigious and financially secure. Consequently, an insufficient number of trained staff is in attendance in pharmacies during opening hours. Participants’ quotes below illustrate this situation:

*“Now I only have one pharmacy technician. A year ago, I had four and then all of them got accepted as government employees. One of them is still working in this town, so she still can work here at night, but the other three work in other towns. We have tried to look for replacements but we could not find one*.” (pharmacist)

*“No pharmacist comes to [this province] without having a main goal of becoming a government employee. Working at a government office is more promising for their future because it gives more secure income compared with [working] in the private sector. Although pharmacies are health service providers, they are still private. There is no salary standard, thus there is no income security in working as pharmacists in pharmacies. Besides, don’t forget about our culture, there is prestige in somebody becoming a government employee.”* (pharmacist)

#### Time for counselling

As a consequence of what was perceived by participants as an insufficient number of trained staff working during pharmacy opening hours, pharmacists found themselves having to handle everything and thus complained that they did not have time to counsel patients with self-medication requests. One pharmacist stated: *“The time to serve patients is limited. There are only two of us at the pharmacy [at which I work] and I have to multitask everything, including compounding.”* Another pharmacist’s remarks supported this perspective: *“If it happens to be rush hour at the pharmacy, certainly I cannot counsel patients. I have to move between the front [the counter area] and the back [the dispensing area]. If I don’t help my staff at the dispensing area, we wouldn’t have time to process the prescriptions.”*

#### Staff affordability

The ability of pharmacies to offer competitive salaries to pharmacists or even afford to employ one at all varied depending on pharmacy income. Large pharmacies attached to busy doctors’ clinics have more patients coming in to fill prescriptions than small pharmacies not so attached, and thus generate larger incomes. This gives large pharmacies a greater ability to afford to employ pharmacists and, further, to pay them competitive salaries. A pharmacist manager from a small pharmacy said she could not afford to employ more than one pharmacist: *“Even to afford one pharmacist’s salary is already difficult.”* The demographic backgrounds of participants, however, seemed to influence their opinions on this issue: only pharmacy owners and/or managers—and not pharmacists and/or other pharmacy staff—remarked upon staff affordability.

#### Facilities to provide consultation

Some participants attributed the lack of counselling in self-medication consultations to a lack of space dedicated for the purpose. A private or semi-private counselling area was considered by some pharmacists as necessary for the ideal provision of consultations for self-medication requests. A pharmacist noted: *“We need a dedicated space; it doesn’t have to be a room. [But] even better if there are chairs and a table to facilitate communication with the patient.”* Again, however, the demographic backgrounds of participants influenced their responses: only pharmacists and/or other pharmacy staff—and not pharmacy owners—reported this issue.

### External pharmacy environment

Our study identified the external factors influencing the provision of self-medication consultations to be: the number of trained pharmacy staff in the research setting; the relevance of pharmacy education to the needs of pharmacy practice; support offered by the Indonesian Pharmacists Association (*Ikatan Apoteker Indonesia*—*IAI*) to local pharmacists; an especially competitive local business environment; and the policy environment.

#### The relevance of pharmacy education to the needs of pharmacy practice

There was a perception among pharmacists that the topics taught at the university lack relevance to everyday pharmacy practice. This lack of relevance appears to be closely related to the knowledge and competence of pharmacists in providing professional self-medication consultations. Pharmacists did not believe that what they learned at university was sufficient to enable them to provide quality patient consultations. One pharmacist reported his university experience as follows: “*at university we learned about pharmacology, pharmacognosy, pharmacy analysis, pharmacy technology, the law, [and] we talked about making drugs. Whereas the topic on how to counsel patients was lacking.”* Another pharmacist reported a similar experience: “*At [pharmacy] school, we were rarely trained in counselling. We only learned the theory, counselling theory, but the practice was lacking.”* Consequently, many pharmacy staff had turned to self-learning to improve their competence for work in the pharmacy. A pharmacist mentioned: “*It is clear that we didn’t learn much about [counselling] from our lectures, so we tend to learn by ourselves.”* Another pharmacist agreed: “*We are self-taught people, we learn after we graduated.”*

#### Support from the Indonesian Pharmacists Association (Ikatan Apoteker Indonesia—IAI)

Pharmacists expected that the IAI should play a role in improving the professionalism of pharmacy staff, but believed it was failing in its duty. A pharmacist mentioned: “*They [the IAI] rarely organise seminars here. The IAI actually should organise seminars for us more often, shouldn’t they?”* Another pharmacist stated: “*If the IAI really cares about the current situation, they should not just give us rules. They should provide us with actual support that we can apply in our practice.”*

#### Competitive business environment

Pharmacy owners and/or managers perceived that too many pharmacies were operating within the setting, creating an overly competitive business environment that negatively affected their income, as illustrated below:

*Pharmacy owner : Right now pharmacies are like mushrooms, aren’t they?*

*Researcher : So do you mean pharmacies are too numerous? Does that cause competition?*

*Pharmacy owner : Yes.*

*Researcher : So, you mean that [‘currently’ or ‘at the present time’] doing pharmacy business [profitably?] is difficult?*

*Pharmacy owner : Too many pharmacies. Recently there was a pharmacy over there, but now it is closed down, isn’t it? Why? Because no [i.e., insufficient] income.*

Pharmacies unable to generate and maintain a stable income would struggle to remain viable, further decreasing their ability to afford trained pharmacy staff, and hence provide quality self-medication consultations.

#### Policy environment

Participants reported deficiencies in the implementation of Indonesian Government Regulation no 51 of 2009 (“*Peraturan Pemerintah no 51—PP 51*”). Regulation PP 51 requires that a pharmacist be available [at all times] during a pharmacy’s opening hours and that only pharmacists handle tasks related to pharmaceutical products. Participants ascribed deficiencies in the implementation of PP 51 to: the lack of enforcement by the responsible government agency; the conflicts of interest arising from enforcement officers also being employed in pharmacies (dual employment); and the lack of synchronisation between regulation/policy and the situation in practice.

Some participants commented on the lack of enforcement of PP 51 in the research setting. A pharmacist noted: *“The regulation [PP 51] is there, but the control is just in the organisation [the IAI] and the sanction is weak. It is only an ethical sanction, and thus [it can be regarded that] pharmacists’ ethics are low. However, if [a pharmacist says] my licence has been revoked by the organisation [the IAI] because I rarely come [to the pharmacy], it could be a different story.”*

The lack of enforcement of PP 51 is partly attributable to the conflict of interest likely created by so many pharmacists also working in government offices. While PP 51 requires pharmacies to have a pharmacist available during pharmacy opening hours, the majority of pharmacists are also government employees who cannot always be available during these hours. A pharmacist who also works for the government highlighted the potential for this conflict of interest to occur: *“On the one hand, in the evening, our job is at the pharmacy. On the other hand, in the morning, we are government employees [and] we are the guardians of the law [inspectors]. If, for example, we put forth now [at the pharmacy] that we are the guardians of the law, it means… the pharmacy here would certainly be closed down.”*

Some participants, however, did not consider it possible in practical terms to actually enforce the PP 51 since the regulation was made without taking into account the realities of pharmacy practice. A pharmacist manager reflected: *“How can the regulation be enforced when we have a shortage of pharmacists in this area?”* Another pharmacist agreed: *“When they [the government] made the regulation [PP 51], I think they had considered [the nation’s] workforce. However, they did not look at the conditions in each area. For areas where there is a university producing pharmacists, it is not a big problem to apply the regulation. However, it is hard for areas like ours. We do not have universities that produce pharmacists.”*

## Discussion

This is the first study to explore the factors that influence the practice of self-medication consultations in community pharmacies in a less-developed area of Indonesia. Previous research has reported that the performance of pharmacy staff when handling self-medication consultations in Indonesia is poor [5–7]. However, no published research has investigated the reasons for this poor practice, an understanding of which will be critical for formulating appropriate interventions to improve the situation. The aim of this research is to fill this gap by identifying these factors. This study identified three inter-related themes: (1) pharmacy staff-patient interactions; (2) the organisational context of the pharmacy; and (3) the external pharmacy environment.

The factors identified are complex and inter-related; many internal factors, for instance, are influenced by external factors beyond the control of pharmacy staff. For example, the lack of knowledge and competence of pharmacists that compromises their capacity to provide quality consultations is influenced by the omission of relevant topics from pharmacy school curricula and a lack of support from the IAI. Furthermore, the non-availability of pharmacists during opening hours and pharmacists’ dual employment appear to be the consequences of an insufficient number of pharmacists in this research site [17]. Finally, the ability of pharmacies to afford competitive salaries and supporting facilities is affected by a competitive business environment. Therefore, in order to improve the quality of self-medication consultations in this setting, changes must be made at multiple levels both within the pharmacy and externally.

Studies in developing countries that specifically explore the factors influencing the provision of self-medication consultations in community pharmacies are limited [27, 28]. Consequently, it is necessary to discuss our findings within the broader literature related to pharmacy services more generally (including dispensing practices, pharmaceutical care, and the appropriateness of prescribing by pharmacy staff) [27–36]. Many factors associated with the provision of self-medication consultations identified in this study are similar to those found for more general pharmacy services in other developing countries. For example, patients’ responses were reported in a Vietnamese study to influence the provision of quality self-medication consultations [27]. A lack of knowledge and skills that prevents pharmacy staff from providing quality self-medication consultations and an inadequate standard of pharmacy education have been stated as factors in studies from Vietnam, Brazil, Guatemala, and Mexico [27, 28, 31]. Lack of time and private areas in which to conduct counselling were mentioned in studies related to the provision of pharmaceutical care from China and Jordan [30, 32]. External factors such as a shortage of trained pharmacy staff [29], a lack of synchronisation between regulation/policy and the reality of pharmacy practice [33], and a lack of support from local pharmacists’ associations have also been noted [32]. Despite the identification of multiple factors that have an impact upon the provision of quality self-medication consultations and other pharmacy services, systematic reviews [37, 38] have shown that the majority of interventions conducted in developing countries have targeted the knowledge and skills of pharmacists and/or other pharmacy staff, without taking other factors into account. Only a few studies included regulatory intervention as a means to improve practice [39–41]. While such interventions as have been implemented resulted generally in positive improvements in knowledge and practice, such improvements may not be sustainable over time, nor change the overall quality of pharmacy services including self-medication consultations [37].

This study identifies several factors that are important in shaping the operation of pharmacies and the performance of pharmacy staff when providing self-medication consultations. There appears to be a range of opportunities for intervention. Efforts to increase the number of trained staff in this setting could include: attracting pharmacists and pharmacy technicians from other areas of Indonesia; increasing the number of local pharmacy technician graduates; or providing scholarships enabling students from this area to enrol in pharmacist programs in universities in other areas of Indonesia, with such students then being compelled to work in the research site after graduation. In the meantime, providing basic training for non-trained staff regarding self-medication counselling may be needed for the benefit and safety of patients. This approach has been tried in other developing countries with positive results [42].

A sound financial position is crucial in enabling pharmacies to afford the staff and facilities needed to maintain quality services. In Indonesia, pharmacies are private businesses without government funding and, therefore, pharmacy income is mostly derived from the sale of pharmacy products. In this setting, some pharmacies have an unstable income stream and one of the factors causing this is an overly competitive business environment [36]. The government may need to assess the impact of this competitive business environment and then take appropriate action based on this assessment.

To enable pharmacy staff to provide professional consultations, there appears to be scope for improvement in their knowledge and competence, including cultural competence. This should involve pharmacist and pharmacy technician educational institutions, as well as the professional organisation (i.e., the Indonesian Pharmacists Association). To our knowledge, no research in Indonesia has been carried out in examining these institutions, however, current pharmacy curricula indicate that there can be variations in how the topic of self-medication consultation is taught and assessed among Indonesian pharmacy schools [43, 44]. As a result, there can be variations in the knowledge and skills across pharmacy graduates depending on the school they attended. Standardisation of pharmacy curricula and assessment particularly for the topic of self-medication consultation across pharmacy schools is recommended. The Indonesian Pharmacists Association could also take the lead in providing continuing education to assist pharmacists in their professional development. Further research focusing on the quality of the pharmacy education system and the role of the Indonesian Pharmacists Association regarding the provision of self-medication consultations in community pharmacies is needed.

In addition, while the Standard Operating Procedure (SOP) for providing self-medication consultations in community pharmacies has been established by the Department of Health [26], none of our study participants were aware of or had read this SOP and as such it is not implemented. In addition to problems with implementation, this SOP cannot be considered comprehensive when compared to the statements from the World Health Organization (WHO), the International Pharmaceutical Federation (FIP), and pharmacy textbooks regarding pharmacists’ role in self-medication [1, 45–50]. For example, the information-gathering section of the SOP does not include the asking of questions related to patient medical history and current medications being used. Furthermore, this SOP does not give clear guidance on when patients should be referred to doctors. The guideline that a medical referral should be made when symptoms persist beyond 3 days after performing self-medication has been generalised to apply to all conditions; such advice appears arbitrary as each condition actually has a different timing for medical referral. While a new standard of pharmacy service was published in 2014, it did not include any guidelines for self-medication consultations [51]. Therefore, there is a need to revise the current SOP and then develop appropriate strategies for its implementation.

Finally, patients’ responses to attempts by pharmacy staff to engage them in consultations appear to influence the amount of information that is gathered and provided by pharmacy staff. Engaging patients during the consultation process was perceived to be a challenge in this setting. Patients’ awareness and trust of the role and competence of pharmacy staff, types of requests made by patients, and patients’ cultural characteristics were perceived by pharmacy staff as the factors that influenced patients’ responses. Similar findings have been reported in the literature [52–58]. A public campaign might be useful to raise patients’ awareness of the role of pharmacists, improve patients’ trust of pharmacists; and instil expectations of and acceptance of the need for consultations [59]. At the same time, measures to improve the professionalism of pharmacists need to be carried out. For example, the Indonesian Pharmacists Association could provide training to improve the knowledge, cultural awareness, communication skills, and self-efficacy of pharmacy staff. Such training would assist pharmacists in overcoming the challenges of engaging with patients and could have the additional benefit of raising patients’ confidence in pharmacists [56, 58, 60]. Further research to explore patients’ perceptions and behaviours in relation to self-medication consultations in community pharmacies in this research site is needed.

### Rigour and limitations

The rigour of this study was ascertained by several measures commonly used in qualitative research [61]. Purposive sampling for different participant demographics (including pharmacists, pharmacy technicians, pharmacy owners, and counter attendants) and member checking (a process to confirm findings with the participants) were used to ensure the rigour of the study. Transferability of the findings to other settings in Indonesia may be possible as several aspects related to pharmacy characteristics, health-care system, and pharmacy education system are similar within Indonesia. These findings may also be transferrable to other developing countries as comparison of these findings with literature from developing countries showed similarities [27, 28, 31]. A rich description of the research site was provided for readers to assist judgement regarding which particular aspects of our findings that could be adopted in other settings.

A potential limitation of our study is that only one investigator performed the coding. During analysis, however, regular discussion with co-authors regarding emerging codes and themes was carried out. Furthermore, member checking with participants was undertaken to gather feedback on our interpretation of the data. Another potential limitation to our findings is that they are derived from the perspectives of pharmacy staff whereas, in reality, a range of other stakeholders (including patients, pharmacy schools, professional bodies, and policy makers) influences the provision of self-medication consultations in community pharmacies. Further research involving a wider range of participants would enrich and could confirm our findings.

## Conclusion

Factors influencing the current practice of pharmacy staff when providing self-medication consultations in this setting relate primarily to three main themes: pharmacy staff-patient interactions; the organisational context of the pharmacy; and the external pharmacy environment. The factors identified are complex and inter-related; and therefore multiple strategies addressing these factors are needed to improve current practice of pharmacy staff in the research setting when providing self-medication requests.

### Ethics, consent to participate, and consent to publish

The Human Research Ethics Committee of the University of Western Australia and the local Indonesian Pharmacists Association approved the study. Written, informed consent for participation and publication has been provided by the participants. The name of the city where this study was undertaken was not provided to protect the site.

### Availability of data and materials statement

The datasets supporting the conclusions of this article are available upon requests from the first author (email: cecilia.brata@gmail.com)

## Additional file

Additional file 1: Interview guide. (DOCX 15 kb)

## References

[1] The role of the pharmacist in self care and self medication [http://apps.who.int/medicinedocs/pdf/whozip32e/whozip32e.pdf]. Accessed 7 May 2016.

[2] Statistical Yearbook of Indonesia 2015 [http://www.bps.go.id/website/pdf_publikasi/Statistik-Indonesia-2015_rev.pdf Indonesia 2014]. Accessed 7 May 2016.

[3] Riset Kesehatan Dasar 2013 [http://dinkes.bantenprov.go.id/upload/article_doc/Hasil_Riskesdas_2013.pdf]. Accessed 7 May 2016.

[4] Private sector health care in Indonesia [http://pdf.usaid.gov/pdf_docs/Pnadq842.pdf]. Accessed 7 May 2016.

[5] Ross-Degnan D, Soumerai SB, Goel PK, Bates J, Makhulo J, Dondi N, Sutoto, Adi D, Ferraz-Tabor L, Hogan R: The impact of face to face educational outreach on diarrhoea treatment in pharmacies. *Health Policy and Plann* 1996, 11(3):308-318. 10.1093/heapol/11.3.308 10160376

[6] Puspitasari H, Faturrohmah A, Hermansyah A (2011). Do Indonesian community pharmacy workers respond to antibiotics requests appropriately. Trop Med Int Health.

[7] Himawati ER, Puspitasari HP, Sukorini AS, Hasanah F, Laksono BS, Martin R, Ekawati Z (2011). History taking profile on self medication services of diarrhoea patients at pharmacies in Surabaya. The 2nd International Conference on Pharmacy and Advanced Pharmaceutical Sciences.

[8] Brata C, Marjadi B, Schneider CR, Murray K, Clifford RM (2015). Information-gathering for self-medication via eastern Indonesian community pharmacies: a cross-sectional study. BMC Health Serv Res.

[9] Krishnan HS, Schaefer M (2000). Evaluation of the impact of pharmacist’s advice giving on the outcomes of self-medication in patients suffering from dyspepsia. Pharm World Sci.

[10] Hoffmann W, Herzog B, Mühlig S, Fabian HKR, Thomsen M, Cramer M, Fiß T, Gresselmeyer D, Janhsen K (2008). Pharmaceutical care for migraine and headache patients: A community-based, randomized Intervention. Ann Pharmacother.

[11] Bello SI, Bello IK (2013). Community pharmacist impacts on self-medication management among rural dwellers, Kwara state central, Nigeria. Int J Res Dev Pharm L Sci.

[12] Smith F (2004). Community pharmacy in Ghana: enhancing the contribution to primary health care. Health Policy Plann.

[13] Badan Pusat Statistik Kota X: Statistik Daerah Kota X 2014. X:Badan Pusat Statistik Kota X; 2014.

[14] Badan Pusat Statistik Provinsi Y: Y dalam angka. X: Badan Pusat Statistik Provinsi Y; 2014.

[15] Van de Pas R (2010). Human resources for health, opportunities & challenges in the Indonesian province of Y. *Master Thesis*.

[16] Brown I. The territories of Indonesia. London: Routledge; 2009.

[17] Pusat Data dan Informasi Kementrian Kesehatan Republik Indonesia (2013). Ringkasan eksekutif data dan informasi kesehatan provinsi Y 2013.

[18] Harper M, Cole P (2012). Member checking: can benefits be gained similar to group therapy?. Qual Rep.

[19] Braun V, Clarke V (2006). Using Thematic analysis in psychology. Qual Res Psychol.

[20] McLeroy KR, Bibeau D, Steckler A, Glanz K (1988). An ecological perspective on health promotion programs. Health Educ Q.

[21] Bronfenbrenner U (1977). Toward an experimental ecology of human development. Am Psychol.

[22] Golden SD, Earp JAL (2012). Social ecological approaches to individuals and their contexts: twenty years of health education & behavior health promotion interventions. Health Educ Behav.

[23] Crosby RA, Salazar LF, DiClemente RJ, DiClemente RJ, Salazar LF, Crosby RA (2013). Ecological approaches in the new public health. Healh behavior theory for public health: principles, foundations, and applications.

[24] Grice GR, Monson K, Pitlick J, Chereson R, Duncan W, Geslani G, Kilgore K, Pate PB, Pautler H (2013). Developing a professionalism plan. Innov Pharm.

[25] Hammer DP (2000). Professional attitudes and behaviors: the “A’s and B’s” of professionalism. Am J Pharm Educ.

[26] Kementrian Kesehatan Republik Indonesia (2008). Petunjuk Teknik Pelaksanaan Standar Pelayanan Kefarmasian di Apotek. KEPMENKES 1027/MENKES/SK/2004.

[27] Thang DX (2013). An investigation of non-prescription medicine supply in community pharmacies in Hanoi, Vietnam. *PhD Thesis*.

[28] Kroeger A, Ochoa H, Arana B, Diaz A, Rizzo N, Flores W (2001). Inadequate drug advice in the pharmacies of Guatemala and Mexico: the scale of the problem and explanatory factors. Ann Trop Med Parasitol.

[29] Goel P, Ross-Degnan D, Berman P, Soumerai S (1996). Retail pharmacies in developing countries: a behavior and intervention framework. Soc Sci Med.

[30] Fang Y, Yang S, Feng B, Ni Y, Zhang K (2011). Pharmacists’ perception of pharmaceutical care in community pharmacy: a questionnaire survey in Northwest China. Health Soc Care Community.

[31] da Rocha CE, Bispo ML, Alcantara TS, Brito GC, Vieira MJ, Lyra DP (2014). What do Brazilian community pharmacists know about self-medication for minor illnesses? a pilot study in the northeast of Brazil. J App Pharm Sci.

[32] AbuRuz S, Al-Ghazawi M, Snyder A (2012). Pharmaceutical care in a community-based practice setting in Jordan: where are we now with our attitudes and perceived barriers?. Int J Pharm Pract.

[33] Hussain A, Ibrahim MI (2011). Perceptions of dispensers regarding dispensing practices in Pakistan: a qualitative study. Trop J Pharm Res.

[34] Cederlof C, Tomson G (1995). Private pharmacies and the health sector reform in developing countries - professional commercial and highlights. J Soc Adm Pharm.

[35] Herman MJ, Susyanty AL (2012). An analysis of pharmacy services by pharmacist in community pharmacy. Buletin Penelitian Sistem Kesehatan.

[36] Herman MJ, Handayani RS, Raharni R, Siahaan S (2008). Analisis faktor internal dan eksternal yang terkait dengan model pelayanan prima di apotek. Buletin Penelitian Sistem Kesehatan.

[37] Smith F (2009). Private local pharmacies in low- and middle-income countries: a review of interventions to enhance their role in public health. Trop Med Int Health.

[38] Wafula FN, Goodman CA (2010). Are interventions for improving the quality of services provided by specialized drug shops effective in sub-Saharan Africa? A systematic review of the literature. Int J Qual Health Care.

[39] Stenson B, Syhakhang L, Lundborg CS, Eriksson B, Tomson G (2001). Private pharmacy practice and regulation - A randomized trial in Lao PDR. Int J Technol Assess Health Care.

[40] Chuc NTK, Larsson M, Do NT, Diwan VK, Tomson GB, Falkenberg TE (2002). Improving private pharmacy practice: A multi-intervention experiment in Hanoi, Vietnam. J Clin Epidemiol.

[41] Chalker J, Ratanawijitrasin S, Chuc NT, Petzold M, Tomson G (2005). Effectiveness of a multi-component intervention on dispensing practices at private pharmacies in Vietnam and Thailand – a randomised controlled trial. Soc Sci Med.

[42] Mutie MK (2011). A systematic review of the training of health care workers within essential medicines supply programs in developing countries. *Master Thesis*.

[43] Silabus kurikulum inti program pendidikan sarjana farmasi [http://aptfi.subagiyo.com/sarjana-farmasi/silabus/]. Accessed 7 May 2016.

[44] Silabus kurikulum inti program pendidikan apoteker [http://aptfi.subagiyo.com/profesi-apoteker/silabus/]. Accessed 7 May 2016.

[45] Statement of principle self-care including self-medication: The professional role of the pharmacist [http://www.fip.org/www/uploads/database_file.php?id=204&table_id=]. Accessed 7 May 2016.

[46] Blenkinsopp A, Paxton P, Blenkinsopp J (2009). Symptoms in the Pharmacy: A Guide to the Management of Common Illness.

[47] Berardi RR, Ferreri SP, Hume AL, Kroon LA, Newton GD, Popovich NG, Remington TL, Rollins CJ, Shimp LA, Tietze KJ (2009). Handbook of nonprescription drugs: An interactive approach to self care.

[48] Azzopardi LM (2000). Validation Instruments for Community Pharmacy: pharmaceutical care for the third millenium.

[49] Pray WS (2006). Non prescription product therapeutics.

[50] Rantucci MJ (2007). Pharmacists talking with patients: A guide to patient counseling.

[51] Kementrian Kesehatan Republik Indonesia (2014). PERMENKES no 35 tahun 2014 tentang Standar Pelayanan Kefarmasian di Apotek.

[52] McMillan SS, Kelly F, Sava A, King MA, Whitty JA, Wheelera AJ (2014). Consumer and carer views of Australian community pharmacy practice: awareness, experiences and expectations. J Pharm Health Serv Res.

[53] Public awareness of community pharmacy and pharmacist [http://www.mps.org.my/publications/Journal_of_Pharmacy/public_awareness.htm]. Accessed 7 May 2016.

[54] Watson MC, Hart J, Johnston M, Bond CM (2008). Exploring the supply of non-prescription medicines from community pharmacies in Scotland. Pharm World Sci.

[55] John DN, Krska J, Hansford D (2003). Are customers requesting medicines by name less likely to be advised or referred? Provision of over-the-counter H2-receptor antagonists and alginate products from pharmacies. Int J Pharm Pract.

[56] Communication and cultural knowledge in Aboriginal health care [http://www.westerndesertdialysis.com/archives/references/19981000%20Communication_and_Cultural.pdf]. Accessed 7 May 2016.

[57] de Bittner MR, Nichols-English GJ, Berardi RR, Ferreri SP, Hume AL, Kroon L, Newton GD, Popovich NG, Remington TL, Rollins CJ, Shimp LA, Tietze KJ (2009). Multicultural aspects of self-care. Handbook of nonprescription drugs: An interactive approach to self care.

[58] Kaae S, Traulsen JM, Nørgaard LS (2012). Challenges to counseling customers at the pharmacy counter—Why do they exist?. Res Soc Adm Pharm.

[59] Schommer JC (1997). Patients’ expectations and knowledge of patient counseling services that are available from pharmacists. Am J Pharm Educ.

[60] Skellett L (2012). Cultural awareness and cultural safety. Aust Pharm.

[61] Lincoln YS, Guba EG (1985). Naturalistic inquiry.

