# Supplementary material for: Factors influencing the current practice of self-medication consultations in Eastern Indonesian community pharmacies: a qualitative study
Source: BMC Health Serv Res. 2016 May 13;16:179. doi: 10.1186/s12913-016-1425-3 (PMC4866032; doi:10.1186/s12913-016-1425-3)
Supplement: Additional file 1: — Interview guide. (DOCX 15 kb) [file 12913_2016_1425_MOESM1_ESM.docx]

**Additional File 1: Interview Guide**

1. I would like you to think about instances when you are handling symptom-based requests in your pharmacy. For example when a patient asks for treatment for cough, what types of questions do you ask?

PROBE: Why do you think you need to ask these questions?

What about questions, such as ….[provide examples of questions that the interviewee has not already mentioned]. Do you often ask these questions? Why didn’t you ask the questions? Do you think the unasked questions are important and that you ought to ask them?

1. I would like you to think about instances when you are handling product-based requests in your pharmacy. For example when a patient asks for Bisolvon, what types of questions do you ask?

PROBE: Why do you think you need to ask these questions?

What about questions, such as ….[provide examples of questions that the interviewee has not already mentioned]. Do you often ask these questions? Why didn’t you ask the questions? Do you think the unasked questions are important and that you ought to ask them?

[If there are differences on the types of questions asked between the symptom-based requests and product-based requests]: Why do you ask different questions for symptom- based requests from those you ask for product-based requests?

1. When you are about to give advice to your patient, what types of things do you take into consideration in your decision to recommend products or to refer patients to a doctor?
2. What do you think regarding providing information such as non-pharmacological information, or medicine information if you are selling a product?

PROBE :

- Is such information important? Why it is important or not important?
- Do you regularly provide verbal medicine information when selling a product for self- medication?
  - If YES, what types of information do you provide? What about information such as..... [provide examples of medicine information that the interviewee has not already mentioned] Why didn’t you provide this information? Do you think this kind of information is important and that you ought to provide it to patients?
  - If NO, why? PROMPT: knowledge, patients’ responses, etc.; PROBE: ask for examples.

1. I would like you to think about when you are responding to patients with self-medication requests. What makes providing these services difficult? What makes providing these services easy?

PROMPT: patients’ responses, knowledge and skills, etc.;

PROBE: ask for examples.

1. In your opinion, what would be the ideal model for responding to patients with self-medication requests? How does this compare with what you do currently? What do you need to enable you to respond to self-medication requests in the ideal way? PROMPT: training (knowledge, communication skills), workload, etc.
